# Together Forever: Bacterial–Viral Interactions in Infection and Immunity

**DOI:** 10.3390/v10030122

**Published:** 2018-03-10

**Authors:** Zhenda Shi, Andrew T. Gewirtz

**Affiliations:** Center for Inflammation, Immunity and Infection, Institute for Biomedical Sciences, Georgia State University, Atlanta, GA 30303, USA; agewirtz@gsu.edu

**Keywords:** rotavirus, reovirus, LPS, inflammation

## Abstract

Most viruses first encounter host cells at mucosal surfaces, which are typically colonized by a complex ecosystem of microbes collectively referred to as the microbiota. Recent studies demonstrate the microbiota plays an important role in mediating host–viral interactions and determining the outcomes of these encounters. This review outlines recently described examples of how bacteria and viruses impact each other particularly during infectious processes. Mechanistically, these effects can be broadly categorized as reflecting direct bacterial–viral interactions and/or involving microbial impacts upon innate and/or adaptive immunity.

## 1. Introduction: Host–Pathogen Encounters Occur in a Complex Ecosystem

**Figure 1:**
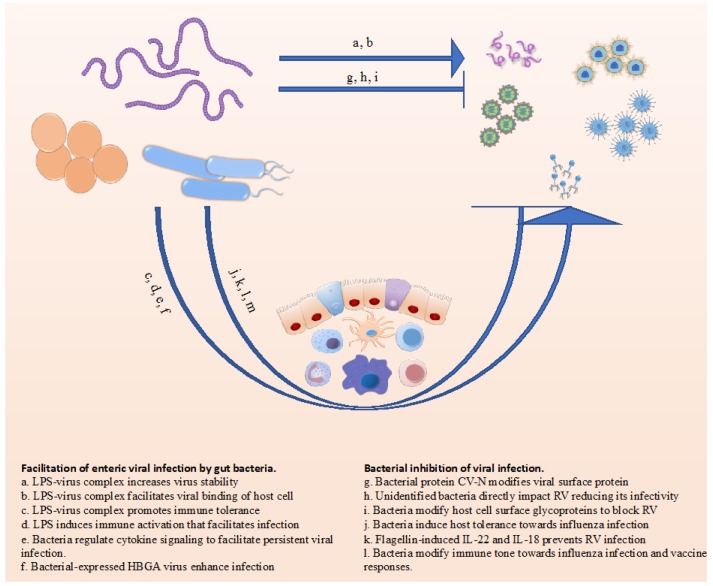
Summary of some of the ways bacteria impact viral infection.

Rigorous application of scientific methods in general, and Koch’s postulates in particular, encourages one to think about and study infectious diseases in reductionist systems that focus on how a potentially pathogenic microbe interacts with host cells. However, in fact, most hosts, especially their mucosal surfaces, harbor complex ecosystems of diverse microbial communities of bacteria, fungi, and viruses, collectively referred to as the microbiota. The specific microbiota composition present during, and even prior to, encounter of potentially pathogenic organisms will often have an extensive influence on whether the pathogen causes a productive and/or symptomatic infection. To date, a large body of work has focused on how the bacterial portion of the microbiota influences an array of disease outcomes, including infectious disease. In comparison, studies or how commensal fungi and viruses might themselves directly influence disease and/or interact with bacteria to do so, has only begun to be appreciated. Herein, we will review some of the recent progress in this latter area, focusing on how bacteria and viruses can directly, and indirectly, influence each other. Many of the findings are summarized in Figure 1. We will focus largely on interactions involving the gut microbiota but will extend discussion into how this system can influence immune responses and infections in other locales.

## 2. Facilitation of Enteric Viral Infection by Gut Bacteria

The broad notion that the microbiota can influence susceptibility to infection has long been appreciated by observations in humans and animal models that antibiotic usage increases susceptibility to numerous bacterial infections, especially well documented for *Clostridium difficile* and *Salmonella* via process termed colonization resistance. Direct dramatic demonstrations of these phenomena include exquisite sensitivity to colitis and death upon challenge of germfree or antibiotic-treated mice upon challenge with these bacterial pathogens. Moreover, it has long been appreciated that the microbiota promotes immune system development and hence, germfree mice would lack a robust immune system, especially in terms of mucosal immunity that plays a key role in many enteric viral infections. Based on these observations, it was presumed that microbiota ablation, achieved by antibiotic or germfree approaches, would result in increased susceptibility to infection of viruses, especially those that enter their hosts via the gastrointestinal tract. However, as outlined below, in contrast to this expectation, lack of a microbiota has been found to result in reduced infectivity for several such viruses including poliovirus, reovirus, rotavirus and mouse mammary tumor virus (MMTV). A range of different mechanisms have been proposed to account for such observations, which can be broadly categorized as primarily reflecting a direct impact of the gut bacteria on the virus or involving immune-mediated effects.

### 2.1. Direct Impact of Microbiota on Virus

Considering that physical/chemistry stability of virions in their between-hosts environment is often a major determinant of their infectivity and that the gastrointestinal tract is a complex, and potentially hostile environment to viruses, one might imagine that some viruses evolve means of utilizing substances in that environment to aid their stability. Indeed, at least one virus, Poliovirus, which can invade the central nervous system and cause paralysis, this is the case. Poliovirus is transmitted via the fecal–oral route, where co-existing microbiota populations are high. Robinson et al. demonstrated that antibiotic treatment before poliovirus inoculation decreased mice susceptibility towards infection by reducing poliovirus replication. Further studies revealed poliovirus particles could also bind to bacterial peptidoglycan and lipopolysaccharide (LPS), which facilities the virus binding on poliovirus receptor of the host. Such studies also demonstrated antibiotic treatment was effective for limiting reovirus infection, suggesting bacteria is facilitating replication and pathogenesis of reovirus, though the detailed mechanism remains unclear [1,2]. Moreover, such peptidoglycan or LPS viral particle complex increased the resistance of poliovirus against environmental challenges such as heat that, otherwise, destroy its infectivity. In contrast, a mutated strain poliovirus, VP1-T99K, which has a reduced binding ability with LPS showed relative instability when added to feces [3]. It seems very reasonable to envisage that analogous mechanisms are used by other viruses that transit the gastrointestinal tract although direct evidence in support of other examples of this notion are not yet in hand.

### 2.2. Dependent on Host Immune System

The notion that intestinal viruses, would bind bacterial products present in the gut lumen is also proposed to govern MMTV infection, albeit by a very distinct mechanism. MMTV is transmitted from mother to offspring via milk. Such vertical transmission is thought to rely upon on activation of toll-like receptor 4 (TLR4), a pattern recognition receptor for bacteria lipopolysaccharide (LPS), which can bind MMTV in the intestine. In mice lacking TLR4, MMTV was no longer transmissible in that a robust cytotoxic immune response efficiently cleared infected cells [4]. Related studies in antibiotic treatment or in germ-free conditions and found that vertical transmission to their offspring was effectively abolished by the production of MMTV specific antibody. Further studies into the mechanism underlying these observations found binding of MMTV-bound LPS complex activated TLR-4/MyD88 pathway and to induce IL-10 that resulted in immune tolerance to MMTV infection [5]. The extent to which other viruses might rely upon similar mechanisms will certainly require further studies. Meanwhile, it has also been proposed that LPS-induced immune activation, rather than suppression, can also promote infection, in particular for Theiler’s murine encephalomyelitis virus (TMEV), which primarily infects mouse strains and causes immune-mediated demyelinating disease. Specifically, Pullen et al. found that when challenging C57BL/6 mice, which are resistance to TMEV, co-administration of the virus with LPS induced many (about 50%) of the hosts to display infected signs of hypersensitivity and proliferation of T cell. Treatment of IL-1β, which was induced by TLR-4 activation also facilitated TMEV infection. It was proposed that LPS induced a hyper-inflammatory condition in the central nervous system that enhanced the relative infectivity of TMEV on the host albeit by a not well defined mechanism [6]. Indeed, direct effects of bacteria and/or their products on innate immunity may be a broad mechanism by which bacteria impact viral infection. Norovirus (NV), which now accounts for about 90% of worldwide incidence of viral gastroenteritis, generally takes longer than 10 days to clear and viral shedding can last for weeks to months by most hosts and, in some cases, can result in persistent infections [7,8,9] Virgin and colleagues group demonstrated that gut bacteria are critical for persistence of murine NV (MNV) strain CR6 infection in the mouse intestine in that treatment of antibiotic prevented the chronic infection of MNV while replenishment of gut bacteria reversed such effect. In the absence of interferon-λ, Ifnlr1, Irf3, and STAT1, antibiotic-mediated bacterial ablation no longer prevented MNV infection indicating the protection was mediated by interferon-λ signaling pathway. Rather, in this scenario, while infection of MNV was reduced, the extra-intestinal spread of MNV was not associated with alterations in gut microbiota populations [10].

Another means by which bacteria enhance norovirus infection is via infecting and trafficking in immune cells. Specifically, a pioneering study from Jones et al. found that NV was able to infect both human and mouse B-lymphocytes in a microbiota-dependent manner [11]. Thus, absence of B-cells or ablation of microbiota reduced MNV infectivity in mice. Infection of human B-cells by NV required the presence of histo-blood group antigen (HBGA)-expressing bacteria. Such findings explained earlier observations that HBGAs impact susceptibility to NV. Specifically, Miura et al. had previously demonstrated that A-like substance (blood antigen type A like substance) of extracellular polymeric substances (EPS), which belongs to the HBGA, is critical for NV and bacteria. Cleavage of *N*-Acetylgalactosamine of EPS significantly reduced the binding of bacteria and NV. Mutation at W375A blocked the binding of NV strain Gl.1 with bacterial EPS [12]. Thus, NV has seemed to utilize a variety of mechanisms to interact with bacteria to promote its infection and persistence.

### 2.3. Rotavirus: Most of the Above, and More

While it is instructive to categorize bacterial modulation of viral infection as being dependent or independent on host immunity, some viruses can be impacted by bacteria by both of these mechanisms. The example with which we are most familiar, in large part because our research focusses on it, is rotavirus. Rotavirus (RV) is a non-enveloped dsRNA virus and is responsible for a major worldwide burden of severe dehydrating diarrhea, causing approximately 200,000 deaths annually, primarily in young children in developing countries [13]. RV primarily infects villous intestinal epithelial cells, which shed the virus into the lumen where it interacts with gut microbiota, which one might imagine might impact its ability to infect new cells with the host and or impact its transit via fecal matter to new hosts. Analogous to the case of poliovirus and reovirus, ablation of microbiota in mice, achieved by use of antibiotics or germfree approaches, reduces initial infectivity of RV. However, the underlying mechanism has remained elusive. It is tempting to imagine analogous mechanism to reovirus and poliovirus, but RV is an even more stable virus as exemplified by the combined need for UV radiation and psoralen to render it non-infectious. Moreover, incubation of RV with LPS did not alter its infectivity in vitro or in vivo. Nonetheless, we speculate some as yet undefined bacterial component may facilitate RV binding and or internalization. In any case, microbiota can also impede RV infection by impacting adaptive immunity. Specifically, microbiota ablation also results in more robust immune responses to RV as evidenced by numbers of anti-RV IgA+ positive plasma cells and fecal and serum anti RV-antibodies. This effect is presumed to be mediated by the microbiota broadly setting the tone of the adaptive immune system such that, in the absence of a microbiota in which the immune system is in a state of minimal activation, sensing of RV results in a readily-detectable signal of immune activation that serves as a robust “signal 2” that drives co-stimulation of antigen-specific lymphocytes in a milieu in which the reduced levels of bacterial antigens would reduce competition for RV antigens. Thus, at least in some scenarios, gut bacteria can promote RV infection by facilitating initial infection and impeding adaptive immunity [14]. However, the extent to which such events happen is very much dependent upon the specific microbiota composition of a particular host. Indeed, we have also observed that some colonies of our mice are highly resistant to RV infection and such resistance it can be transferred to new hosts via fecal microbial transplant. Such protection is independent of adaptive immunity in that can be observed in Rag1-deficient mice, which lack functional T- and B-cells. We are currently working to identify the specific microbes that mediate such protection against RV and define the underlying mechanism of action but submit that, in any event, this observation highlights the notion that the extent to which bacteria promote and/or impede viral infection will vary in different hosts in part based on microbiota composition.

## 3. Bacterial Inhibition of Viral Infection

Multiple counterpoints can be envisioned to aforementioned notion that viruses that infect via the gastrointestinal tract would have learned to use bacteria and/or their products to promote viral infection. One would be that the mammalian intestine in adapting to survive amidst the large microbial load it harbors, would have selected for bacteria that might, somehow, protect from various infections, including by viruses. Another would be that in the highly diverse complex intestinal environment, which microbes produce a far greater array of molecules than that produced solely by the host, that there are likely to be some products with some sort of antiviral activity. Indeed, while it is difficult to say which of these perspectives is most relevant, there are certainly several specific examples whereby bacteria and their products impede viral infection.

### 3.1. Bacteria Can Directly Interact with Virus to Reduce Infectivity

Bacteria have long been known to secrete a chemically diverse array of anti-microbial peptides, namely bacteriocins, that have broad and specific activity against various other bacteria. Such bacteriocins are presumed to play a central role in colonization resistance and may play a key role in determining microbiota composition. Given the diverse chemical mechanisms by which such products act, it seems both tempting and reasonable to presume that bacteria might also secrete proteins with direct anti-viral activity. There is indeed one well documented example of this notion. Specifically, Boyd et al. isolated an 11 kDa protein from a culture of *Cyanobacterium Nostoc ellipsosporum*, which was originally recovered from algae, termed cyanovirin-N (CV-N), that has direct antiviral activity against HIV [15] and influenza virus [16]. Native and recombinant CV-N exhibits a high-affinity interaction with HIV envelope protein glycoprotein 120 that interferes with HIV binding of CD4 and correlates with a marked reduction of HIV infectivity in cultured CEM-SS cells. In the case of influenza virus, CV-N appeared to bind hemagglutinin, which has long been appreciated to mediate cellular entry of this virus. While these studies were undertaken with the goal of screening mixtures of compounds to develop novel therapeutic agents to treat these infections, it does not seem to be a great stretch to imagine that such compounds might exist in bacteria-dense milieus such as the distal colon, where initial encounters of many human hosts with HIV occur. Hence, we submit is remains an important goal to screen human microbiota for antiviral products, especially against diseases transmitted via anal sexual contact. From our own work on RV described above, we note that the microbiotas that can confer protection against RV upon transplant, have direct anti-RV activity when incubated with this virus in vitro suggesting the existence of some antiviral agents in the gut microbiota.

Another potential means by which bacteria might impede viral infection is to impact the cell surface molecules to which they bind. Indeed Varyukhina et al. demonstrated that *Bacteroides thetaiotaomicron* and *Lactobacillus casei* can modify cell surface glycoproteins that mediate RV binding. Specifically, they showed that these bacteria and cell-free supernatants therefrom, modified HT-29 cell surface glycan by blocking galactosylation to increase galactose levels and reduce RV infection in vitro. Consequently, the authors suggest that bacterial modification of cell-surface glycans might be used as a means for treating RV infection [17]. It is possible that this mechanism revealed in this in vitro study may underlie the protective effect reported for *Lactobacillus rhamnosus* GG (LGG) on RV-induced disease. Specifically, Guandalini et al. reported that administering this bacteria to children with RV-associated diarrhea significantly shortened the duration of diarrhea on average, as well as reduced long-term diarrhea cases [18]. Thus, while mechanisms need deeper study, bacterial–viral interactions may prove exploitable to ameliorate severe disease.

### 3.2. Bacterial Impacts on Immune System Can Impede Viral Infection

One long generally appreciated means by which bacteria can impact the outcome of infections in general, including viral infections, is by promoting immune development, especially in terms of mucosal immunity. Specifically, it has long been appreciated that raising mice in germfree conditions results in a near complete absence of gut-associated lymphoid tissue (GALT), which is necessary for robust production of mucosal antibodies that are known to be the primary correlate of protection against rotavirus, for example. More recently, some specific examples of how bacteria and their products impact viral infection via impacting innate and adaptive immunity have been worked out in some detail. Wang et al. demonstrated that an upper respiratory tract commensal bacterium, *Staphylococcus aureus*, could rescue the specific pathogen-free mice from death resulting from influenza infection and related inflammation. The mechanism that *S. aureus* uses to provide such protection relies on activation of TLR-2 signaling that drives recruitment of peripheral CCR2 (+) CD11b (+) double positive cells to alveolar and subsequent maturation of these cells into M2 macrophages. M2 macrophages then mediate host tolerance against lethal inflammatory responses upon influenza infection. This study points out the importance of commensal bacterial for priming the host into appropriate tolerance levels for facing viral infection [19].

Our work described that a bacterial product, namely flagellin could provide strong protection against rotavirus. In particular, we reported that systemic treatment of mice models with bacterial flagellin prevented and cured rotavirus (RV) infection [20]. Such protection was not absolutely restricted to RV infection but rather extended to some other enteric viral infections such as reovirus. Mechanistically, flagellin’s antiviral infection relied on activation of both Toll-like receptor 5 (TLR-5) and NOD-like receptor C4 (NLRC-4) and downstream cytokines IL-22 and IL-18 and was independent of interferon (types I, II, and III), which mediate most pathways of antiviral immunity. While the mechanism by which IL-18 and IL-22 work in concert to clear and prevent RV infection remains under investigation, it seems to involve a combination of driving IL-18-induced apoptosis, preferentially of RV-infected cells, and IL-22-induced proliferation that together result in turnover of villus epithelial cells at a rate faster than the rate at which virus can infect new cells. While it may be possible to harness this mechanism to develop therapies to treat RV infection, especially in immune compromised hosts whom can develop chronic RV infections, the extent to which such systemic treatment with flagellin, or IL-18/22 mimics events that might occur during any infectious process is unclear but nonetheless highlights strong theoretical potential for one infection to impact another.

Another compelling demonstration of the ability of commensal gut bacteria to broadly impact immune function during an infection can be found in work from Abt and colleagues. They reported that microbiota ablation via antibiotic treatment markedly delayed mouse clearance of lymphocytic choriomeningitis virus (LCMV) infection. Antibiotic treatment impaired both innate and adaptive innate antiviral responses wherein both CD8+ T cell response and IgG titer in the blood were attenuated in response to LCMV infection. Macrophages isolated from the mice that received antibiotic treatment showed impaired activation upon stimulation of type I and II interferons and consequently impairment of responses that would normally have limited viral infection. These data highlight an important role of commensal bacteria in keeping host immunity in a state ready to be activated to prevent viral infection [21]. Moreover, such setting of the immune system tone can also impact the effectiveness of vaccinations. For example, work from Oh and colleagues found that ablation of gut bacteria via antibiotic or germfree approaches markedly reduced anti-influenza antibodies in response to the trivalent influenza virus vaccine [22]. Such hyporesponsiveness was rescued by colonizing mice with flagellated but not aflagellate *E. coli*. Moreover, mice maintained with a microbiota but lacking TLR5 also exhibited reduced responsiveness to this vaccine thus together suggesting a reliance upon TLR5 by microbiota derived flagellin to maintain the immune system in a state ready to quickly respond vaccines and presumably viral infections [22].

While use of experimental approaches to fully, or near fully, deplete microbiota are useful for examining the general importance of having a commensal microbiota, subtler but potentially broad and important effects will depend upon the specific microbiota composition present. This concept can be seen in work from Moon et al. who reported a mechanism that level of IgA in the GI track was associated with a specific group of bacteria, possibly including Gram-negative fecal anaerobe *Sutterella*, that can be vertically and horizontally transmitted by co-housing or fecal microbiota transplantation [23]. Furthermore, this phenotype was induced by these low IgA associated bacteria, which can degrade fecal IgA and thus lower its levels. Thus, immune tone is not only influenced by microbiota composition but also how such microbiota interact with the mucosal immune system.

## 4. Impact of Viruses on Bacterial Infection

Although not as well understood, or extensively studied as its bacterial component, it is increasingly clear that the microbiome includes an extensive collection of bacteriophages and, to a less well characterized extent, eukaryotic viruses collectively referred to as the virome. Analogous to gut bacteria, the virome is thought to be vertically inherited but modifiable and have a major impact upon host phenotype [24,25]. A recent example of the potential ability of viruses to drive immune development in a manner analogous to bacteria can be seen in the work of Kernbauer and colleagues who found that murine norovirus (MNV) infection could correct many aspects of intestinal and immune phenotype associated with microbiota ablation. Specifically, they observed that the antibiotic treatment, as well as germ-free condition, resulted in the loss of commensal bacterial-induced intestinal abnormities, such as shrinkage in the villus, and an abnormal distribution and function of immune cell population, including over expansion of group 2 innate lymphoid cells (ILCs) in the gut. Addition of MNV to such mice restored a relatively normal gut morphology and immune cell populations and did so in a manner dependent upon type I Interferon. Such correction of immune phenotype by MNV corrected the severe inflammatory pathology in these mice that would have otherwise resulted upon challenge with bacterial pathogen *Citrobacter rodentium* [26] together demonstrating the broad ability of viruses to impact immune phenotype and, consequently, impact the outcome of a bacterial infection. Another striking example virome impacting the immune system is found in the work of Masopust and colleagues who observed that in contrast to mice maintained in modern vivaria, mice captured in the wild and mice purchased from pet stores displayed serologic positivity to many common viruses and had populations of naïve and memory immune cells in roughly equal proportions to that observed in humans. While these common viruses are not classically viewed as being commensal microbes, they might functionally meet this definition in that most hosts are frequently exposed to one or more such agents [27].

Specific examples of the ability of individual viruses to impact bacterial infection in mice with a normal microbiota and immune system can be seen in work from Virgin and colleagues. Barton et al. reported that mice latently infected with either murine γ-herpesvirus 68 (MHV68) or murine cytomegalovirus (CMV), which genetically resemble the human pathogens Epstein–Barr virus and human cytomegalovirus, are resistant to the bacterial pathogens *Listeria monocytogenes* and *Yersinia pestis* [28]. Such protection relies on the systemic activation of macrophages and upregulation of basal innate immune system regarding cytokines production, including the production of cytokine interferon-γ. These observations may reflect that latent infection by these viruses calibrate the host to an immune competent mode thus prime the host for combating the bacterial pathogen challenges [29]. Further studies revealed that the protective activity of MHV68 chronic infection could offset the lethal *Listeria monocytogenes* infection in immunodeficiency host Hoil-1 KO, IL-6 KO, Caspase-1KO and Caspase-1 and Caspase-11 DKO mice by upregulating basal level of innate immune responses, including expression of interferon-γ, suggesting multiple pathways mediate the protection [30].

In addition to impacting infections by individual pathogens, studies on MNV can also be viewed to indicate that viral infection can broadly impact responses to pathobionts and complex microbiotas in general in a manner that impacts disease. Specifically, Cadwell and colleagues observed that the extent to which some mice that are genetically prone to developing microbiota-dependent colitis actually develop disease that can be determined by their carriage of a persistent MNV infection. The immediate immune consequences of persistent MNV infection is to result in modest but chronic elevations in TNFα expression. While WT mice manage to deal with such TNFα expression, in mice with defects in autophagy such as Atg16L1-deficient mice, which cannot efficiently manage intracellular pathobionts, such TNFα expression drives chronic inflammation that manifests as severe colitis [31]. Such observations support the long-standing hypothesis that development of inflammatory bowel disease in patients with a genetic predisposition is triggered by a viral infection and suggest prospective studies should be performed in humans to examine this possibility.

## 5. Perspectives

Herein, we sought to review and discuss how viruses and bacteria might interact in complex environments and, consequently, influence health and disease. We have outlined several examples whereby bacteria and viruses can, directly and/or indirectly, influence each other in the context of both normal development and disease. We submit that some of these examples provide compelling evidence for the importance and potential power of such interactions. Moreover, some of these examples describe tractable models to decipher the molecular interactions that mediate these phenotypic effects. However, at present, the most reasonable overriding conclusion that can be reached is our understanding of these interactions has only begun to scratch the surface. Indeed, further studies in multiple directions are needed, including studies to identify specific examples of bacteria and viruses influencing each other in humans and development of animal and in vitro models that could dissect the molecular underpinnings of these observations.
